# Audiovisual Content for a Radiology Fellowship Selection Process During the COVID-19 Pandemic: Pilot Web-Based Questionnaire Study

**DOI:** 10.2196/28733

**Published:** 2021-05-20

**Authors:** Ivan Rodrigues Barros Godoy, Lus Pecci Neto, Abdalla Skaf, Hilton Muniz Leo-Filho, Toms De Andrade Loureno Freddi, Dany Jasinowodolinski, Andr Fukunishi Yamada

**Affiliations:** 1 Department of Radiology, Hospital do Corao and Teleimagem So Paulo Brazil; 2 Department of Diagnostic Imaging, Universidade Federal de So Paulo So Paulo Brazil; 3 ALTA Diagnostic Center (DASA Group) So Paulo Brazil

**Keywords:** audiovisual reports, COVID-19, fellowship, radiology, smartphones, video recording, web technology

## Abstract

**Background:**

Traditional radiology fellowships are usually 1- or 2-year clinical training programs in a specific area after completion of a 4-year residency program.

**Objective:**

This study aimed to investigate the experience of fellowship applicants in answering radiology questions in an audiovisual format using their own smartphones after answering radiology questions in a traditional printed text format as part of the application process during the COVID-19 pandemic. We hypothesized that fellowship applicants would find that recorded audiovisual radiology content adds value to the conventional selection process, may increase engagement by using their own smartphone device, and facilitate the understanding of imaging findings of radiology-based questions, while maintaining social distancing.

**Methods:**

One senior staff radiologist of each subspecialty prepared 4 audiovisual radiology questions for each subspecialty. We conducted a survey using web-based questionnaires for 123 fellowship applications for musculoskeletal (n=39), internal medicine (n=61), and neuroradiology (n=23) programs to evaluate the experience of using audiovisual radiology content as a substitute for the conventional text evaluation.

**Results:**

Most of the applicants (n=122, 99%) answered positively (with responses of agree or strongly agree) that images in digital forms are of superior quality to those printed on paper. In total, 101 (82%) applicants agreed with the statement that the presentation of cases in audiovisual format facilitates the understanding of the findings. Furthermore, 81 (65%) candidates agreed or strongly agreed that answering digital forms is more practical than conventional paper forms.

**Conclusions:**

The use of audiovisual content as part of the selection process for radiology fellowships is a new approach to evaluate the potential to enhance the applicants experience during this process. This technology also allows for the evaluation of candidates without the need for in-person interaction. Further studies could streamline these methods to minimize work redundancy with traditional text assessments or even evaluate the acceptance of using only audiovisual content on smartphones.

## Introduction

Fellowship programs in radiology are usually 1- or 2-year clinical trainings in a subspecialty area after completion of a 4-year residency program. These fellowships therefore represent an optional sixth and seventh year of clinical training, although this may vary in different countries. Most radiologists trained in the United States complete a fellowship before formally entering practice. In a survey from 1999, 80% of fourth-year and 84.6% of third-year trainee respondents had accepted or were expected to accept fellowship offers [1]. In a survey from 2009, 93.4% of senior resident respondents planned to pursue fellowships [2]. Fellowship trainees often believe that they are less competitive in the job market without a fellowship, and that they may have an advantage in seeking subsequent employment in the same geographic region as that of their fellowship [3]. Starting salaries have also been noted to be low for residency-only graduates [4]. Furthermore, the selection process of the applicants could vary in different countries and institutions. Recent fellows appear to be more satisfied with their selection and application process than their program directors [4]. This study aimed to investigate the utility of audiovisual content as a part of the applicant selection process through the use of the applicants smartphones. The applicants experiences and perceptions with digital forms and questions were evaluated in comparison with traditional paper-printed tests currently used as the evaluation method in medical school and during radiology residency in the country where this study was performed.

The current literature contains little information regarding the audiovisual content of radiology studies, especially regarding fellowship candidate selection methods during the application process [5,6]. Modern web-based technology and screen capture software allow for the development of an environment where audiovisual files can be easily created and shared for clinical and educational purposes, using cloud technology.

The COVID-19 pandemic has evolved rapidly in most countries and widely disrupted personal and professional lives, having also affected the process of selecting radiology fellows and radiology education [7,8]. In this study, audiovisual content using smartphones was used as a supplemental material for the radiology fellowship selection process. The aim of this study is to evaluate candidates experience in using audiovisual content with their own smartphones, especially as an alternative method of evaluation during the COVID-19 pandemic while maintaining social distancing.

## Methods

**Figure 1:**
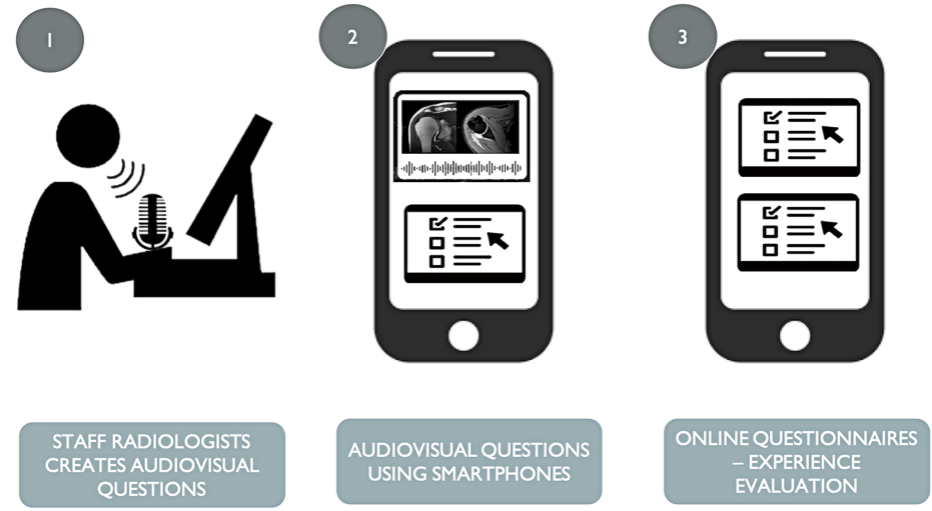
Summary of the steps of the workflow of this study.

This study was approved by the institutional review board of the participating institutions and was compliant with the guidelines of the Health Insurance Portability and Accountability Act of 1996. Informed consent was waived for participants included in the study after institutional review board approval. Our study used a 3-step approach (Figure 1).

**Step 1:** one radiology staff member of each specialty (musculoskeletal, internal medicine, and neuroradiology) generated 4 audiovisual questions, each referring to radiology cases from institutional records. These audiovisual questions were generated using Screencast-O-Matic screen capture software (version 3.8.0, Screencast-O-Matic) in a personal password-protected computer from the hospital. A standard radiology workstation dictaphone was used for audio recording. Videos were saved in MP4 format and uploaded to the institutions picture archiving and communication system using the softwares application programming interface in accordance with the guidelines of the Health Insurance Portability and Accountability Act of 1996 with interoperability via HL7. This study included typical cases such as a bucket handle meniscal tear, subdural hematoma, and appendicitis, with a total of 6 questions in the audiovisual format. The cases included in the questions were anonymized using a built-in hot-key feature of the picture archiving and communication system to prevent the release of personal information contained in the radiology cases.

**Step 2:** the candidates received a web-based questionnaire (Google Forms) via email, which contained 4 audiovisual questions with multiple-choice answers for the subspecialty the candidate applied for. Each correct answer was automatically computed in the candidates profile, and upon completion of the test, all the participants received an updated ranking of the evaluation via email.

**Step 3:** The candidates answered a final web-based questionnaire about their experience with using their own smartphones to access the test with questions in the audiovisual format. The questionnaire included questions to measure concordance with a Likert-type scale, with the exception of the question on the operating system of the smartphone and one regarding the use of earphones. The scoring system was based on a 5-point scale with scores ranging 0-4, where 0=totally disagree, 1=partially disagree, 2=neither agree nor disagree, 3=partially agree, and 4=totally agree.

The questions of the second questionnaire were as follows: (1) I would like to view my score immediately after the test is over; (2) I am used to watching audiovisual content on my smartphone; (3) I prefer to answer questions on traditional paper instead of the digital form; (4) answering digital forms is more practical than conventional paper forms; (5) images in digital forms have superior quality than printed in paper; (6) I feel safer answering in printed text than in digital forms; (7) the presentation of the cases in an audiovisual format facilitates the understanding of the findings; and (8) I felt in disadvantage due to the screen size of my smartphone.

The generation of the audiovisual radiology questions lasted <5 minutes for each case once each radiologist was familiar with the screen capture software. The purpose of the videos is to reflect the radiologists viewpoint in each case, including the sequences used to evaluate the findings and pointing to relevant alterations (Multimedia Appendix 1). Each audiovisual question comprised a video of <2 min, ranging in size from 2 to 12 megabytes. Those videos were uploaded in MP4 format to the web-based questionnaire (a Google Form) with the respective question and multiple-choice answers. All questions were sent to the applicants via email and contained a password-protected weblink. The candidates were instructed to open the questionnaires on their own smartphones and watch the audiovisual questions, using earphones for better audio quality.

The results are summarized using simple and relative (percentages) frequencies and represented by bar graphs and pie charts. The Fisher exact test was performed to analyze the associations between the questions and the candidate groups. Data graphics were produced using Microsoft Excel. Data analysis was performed using the R statistical program for Windows (The R Foundation) using the Rcmdr package and RStudio platform.

## Results

The mean age of the candidates was 30.1 (SD 2.6) years, and the mean period since their graduation from medical school was 5.4 (SD 2.2) years. Most of the applicants smartphones had an iOS operating system (n=77/98, 78.6%), and the remaining had Android smartphones. This difference was not significant among candidates of musculoskeletal, internal medicine, and neuroradiology subspecialties (*P*=.38).

Regarding the use of smartphones to watch any type of audiovisual content, most of the candidates answered that they frequently use their own device (n=77/123, 62.6%) and also using earphones for better audio quality (n=108/123, 87.7%). These findings are not significantly different among the 3 radiology subspecialties (*P*=.88).

To the question, I would like to view my score immediately after the test is over, most of the applicants responded with strongly agree (n=94/123, 76.4%), although there was a significant difference among the 3 subspecialty groups where 51/61 (83.6%) strongly agreed in the internal medicine group, 30/39 (76.9%) in the musculoskeletal group, and 13/23 (56.5%) in the neuroradiology group (*P*=.02).

To the question, I feel safer answering questions in printed text than in digital forms, most of the candidates responded with neutral (n=36/98, 36.7%). There was a significant difference in responses among the 3 subspecialty groups, with 8/19 (42.1%) of the internal medicine applicants, 3/32 (9.4%) of the musculoskeletal applicants, and 18/47 (38.3%) of the neuroradiology applicants responding with agree (*P*=.04).

The answers to the other questions were not significantly different among the radiology subspecialty groups (*P*>.05). Our findings regarding the responses from all candidates are summarized in Table 1. The great majority of applicants (n=122, 99%) agreed or strongly agreed that images in digital forms have superior quality to those printed on paper. In total, 101 (82%) applicants concurred with the statement that the presentation of the cases in audiovisual format facilitates the understanding of the findings. Furthermore, most candidates agreed or strongly agreed that answering digital forms is more practical than answering conventional paper forms (n=81, 65%).

**Table 1:** Distribution of questionnaire responses from all candidates (N=123).

Total	Strongly disagree	Disagree	Neutral	Agree	Strongly agree
Images in digital forms have superior quality than printed in paper, n (%)	0 (0)	1 (1)	0%	49 (40)	*73 (59)^a^*
The case presentation in audiovisual format facilitates the understanding of the findings, n (%)	0 (0)	6 (5)	16 (13)	*65 (53)*	36 (29)
I feel safer answering in printed text than in digital forms, n (%)	7 (6)	15 (12)	*46 (37)*	37 (30)	18 (15)
Answering digital forms is more practical than in conventional paper forms, n (%)	4 (3)	21 (17)	17 (14)	*54 (43)*	27 (22)
I prefer to answer questions on traditional paper instead of this digital form, n (%)	10 (8)	27 (22)	44 (35)	22 (18)	20 (16)
I felt unfavored due to the screen size of my smartphone, n (%)	21 (17)	39 (32)	*41 (33)*	22 (18)	0 (0)

^a^Italicized values represent the preferred answer.

## Discussion

### Principal Findings

This study was focused on the experiences of users with audiovisual content in digital questionnaires and not on the answers to the radiology questions that the candidates ranked by themselves. Most of the answers regarding the experience with this technology were positive, especially those suggesting that digital forms are more practical than conventional paper forms, radiology images and videos have superior quality than those printed on paper, and the presentation of the cases in an audiovisual format facilitates the understanding of imaging findings. These findings suggest that the adoption of this technology may increase the perception of quality during the selection process, especially during the COVID-19 pandemic.

During the last few years, little progress has been made in the format of the selection process of radiology fellows. The process usually varies from country to country and even among different programs in the same city. Program directors usually include traditional tests printed in paper, curriculum analysis, and interviews for a candidates selection. In our institution, the fellows are selected on the basis of a multiple-choice test printed on paper, often in a spacious room with capacity of 200-300 people. After the printed test, the applicants are divided in 3 groups, namely musculoskeletal, internal medicine, and neuroradiology, for curriculum analysis and interviews. The ranking of the candidates is later publicized for all the participants.

New challenges have emerged from this pandemic, mostly regarding how to balance activities as close to normal as possible and following all security measures. A recent study proposed measures to maintain radiology education during the COVID-19 pandemic, including the use of web-based platforms constantly with case-based teaching, with read outs that can be attended over the internet and with screen sharing and chats [7]. Furthermore, virtual rounds with multidisciplinary case discussions and weekly article discussions are interesting approaches to preserve the feeling of normalcy [8]. Another study by Chong et al [9] suggested the development of a specific plan in response to the pandemic to ensure the safety and well-being of the radiology trainees. Those measures should include redistribution of work based on the clinical demand and pandemic status, promoting social distancing by reducing the number of radiologists in each rotation and reading rooms, using personal protective equipment for patient and staff protection, and maintaining radiology teaching using web-based platforms [9].

Audiovisual content using screen capture software is a promising tool with few reports in the literature, with applications in research and academia [10] and recently described as a technology to enhance traditional text reports of emergency musculoskeletal cases [6]. Videos narrated by the radiologist showing imaging findings have the potential to generate high-quality content useful for education and facilitate the understanding of imaging studies for the ordering physicians [6].

The dedicated audiovisual content in this study was focused on enhancing the experience of candidates during the selection process to simulate the evaluation of an actual case through narrated videos. Live or recorded audiovisual material may be used to increase communication between physicians and radiologists and may also be used as a teaching platform for case conference presentations and clinical rounds [6,10]. This technology could also enable physicians to better explain imaging findings to their patients on handheld devices, such as smartphones and tablets [10].

Social restrictions have been imposed during the COVID-19 pandemic, such as those on face-to-face clinical consultations and the increased use of alternative technologies such as telemedicine and the use of smartphones [11]. Studies have reported the successful use of smartphones for fracture diagnosis in musculoskeletal trauma cases [12] and for the identification of pediatric supracondylar fractures [13]. In particular, 5G smartphone technology is a step forward in connection speed and efficiency, with the potential to facilitate web-based interactions as close to in-person activities, including patient consultations, monitoring, and high-speed data file transfer, including imaging studies [14]. To our knowledge, this is the first study to include smartphones and digital questionnaires with audiovisual content as part of the radiology fellowship selection process; therefore, the potential of this technology is still not fully evaluated.

An unexpected observation of our study was that 29.6% of the candidates indicated that they usually feel safer taking paper-printed tests rather than completing digital forms, and 18.4% felt disadvantaged while answering the questions on their own smartphones owing to the size of the screen. This may be due to an insecurity of the impact of this new technology in the selection process. In our opinion, digital forms containing videos with the radiologist narrating the findings is a great tool to increase the experience of the candidates and approximate the viewer close to a real-time evaluation of cases. Another interesting observation is that most of the interviewed candidates frequently consume audiovisual content on their own smartphones (62.6%). A recent study demonstrated that approximately 59% of adults recently consumed health information on the internet, including social media platforms such as YouTube [15]. Furthermore, radiologic content on social media, usually accessed on smartphones, is an emerging technology with the benefit of reaching larger audiences than traditional educational methods [16]. We speculate that an audiovisual report with medical content meets the patients expectation of a dynamic way of expressing the findings of their imaging studies.

### Limitations

One limitation that was noted during the study is that smartphone screen size and operating systems were not standardized. A bigger screen or even using tablets or notebooks could improve the experience of evaluating the audiovisual content of the questionnaires, but we opted to have our candidates use their own smartphones owing to the familiarity of the user with the device and its functionalities and to simulate the experience of receiving an examination to be evaluated on a smartphone, which is a situation often encountered by radiologists. We encouraged the applicants to use earphones and to rotate the smartphone horizontally for better audio and video quality, but we acknowledge that a bigger screen in notebooks could be better.

Furthermore, the questionnaires have important considerations, such as a limited number of questions (information bias) and a small sample size with a probable selection bias. Another limitation is that candidates may feel as though they are being watched during step 3 of the process, which could affect their behavior, as described by the Hawthorne effect [17]. Even with these limitations, the results show the potential of this new form of radiological fellowship selection. Therefore, these findings can be complemented by studies with a larger sample size and more comprehensive questionnaires.

Based on the data obtained in this study, the web-based questionnaire with audiovisual content using smartphones seems to have potential for the application process of candidates for radiology fellowship programs. There was a good response in terms of agility of evaluation and quality of information passed on to the applicants during the selection process, helping them during their first trimester of 2020 with the beginning of the COVID-19 pandemic.

### Conclusions

This study focused on creating web-based questionnaires with smartphones and audiovisual radiology content as an alternative for the traditional in-person selection process with tests printed on paper. This was a pilot study during the onset of the COVID-19 pandemic when measures have been taken to ensure social distancing and attempt to flatten the contagion curve. This method includes the potential to provide quick results, with the safety of password-protected questionnaires. Our evaluation suggests that audiovisual questions may simulate a real-time evaluation of radiology cases and may improve communication between the program directors and the candidates. The fact that the applicants found the audiovisual content in smartphones easier and faster to understand supports that observation. Further studies are necessary to access the acceptance of this form of the radiology selection process in other medical specialties. Additionally, video technology for interviews or the evaluation of remote procedures as part of the selection process should be included. Continued development of standardized web-based tests and questionnaires may encourage future acceptance.

## Appendix

Multimedia Appendix 1 Example of an online question using audiovisual radiology content.
